# A G613A missense in the Hutchinson’s progeria lamin A/C gene causes a lone, autosomal dominant atrioventricular block

**DOI:** 10.1186/s12979-014-0019-3

**Published:** 2014-11-26

**Authors:** Francesco Villa, Anna Maciąg, Chiara C Spinelli, Anna Ferrario, Albino Carrizzo, Attilio Parisi, Annalaura Torella, Chiara Montenero, Gianluigi Condorelli, Carmine Vecchione, Vincenzo Nigro, Annibale S Montenero, Annibale A Puca

**Affiliations:** ITB-CNR, Segrate, MI Italy; IRCCS Multimedica, Milan, Italy; IRCCS Neuromed - Parco Tecnologico, Pozzilli, IS Italy; Università degli Studi di Roma “Foro Italico”, Rome, Italy; Seconda Università degli Studi di Napoli, Naples, Italy; Università degli Studi di Roma Tor Vergata, Rome, Italy; Istituto Clinico Humanitas, Rozzano, MI Italy; Università degli Studi di Milano, Milan, Italy; Dipartimento di Medicina e Chirurgia, Università degli Studi di Salerno, Via Giovanni Paolo II, 132, 84084 Fisciano Salerno, Italy

**Keywords:** Arrhythmia, Dilated cardiomyopathy, Exome sequencing, Atrioventricular block, Lamin A/C

## Abstract

**Background:**

*LMNA/C* mutations have been linked to the premature aging syndrome Hutchinson’s progeria, dilated cardiomyopathy 1A, skeletal myopathies (such as the autosomal dominant variant of Emery-Dreifuss muscular dystrophy and limb-girdle muscular dystrophy), Charcot-Marie-Tooth disorder type 2B1, mandibuloacral dysplasia, autosomal dominant partial lipodystrophy, and axonal neuropathy. Atrioventricular block (AVB) can be associated with several cardiac disorders and it can also be a highly heritable, primitive disease.

One of the most common pathologies associated with AVB is dilated cardiomyopathy (DCM), which is characterized by cardiac dilatation and reduced systolic function. In this case, onset has been correlated with several mutations in genes essential for the proper maturation of cardiomyocytes, such as the gene for lamin A/C. However, no clear genotype–phenotype relationship has been reported to date between *LMNA/C* mutations and cardiomyopathies.

**Results:**

DNA and medical histories were collected from (n = 11) members of different generations of one family, the proband of which was implanted with a pacemaker for lone, type II AVB. Exome sequencing analysis was performed on three relatives with AVB, and the mutations therein identified validated in a further three AVB-affected family members.

In the initial three AVB family members, we identified 10 shared nonsynonymous single-nucleotide variations with a rare or unreported allele frequency in the 1000 Genomes Project database. Follow-up genetic screening in the additional three affected relatives disclosed a correlation between the lone AVB phenotype and the single-nucleotide polymorphism rs56816490, which generates an E317K change in lamin A/C. Although this mutation has already been described by others in a DCM-affected proband with familiarity for AVB and sudden death, the absence of DCM in our large, AVB-affected family is indicative of genotype–phenotype correlation between rs56816490 and a familial, autosomal dominant form of lone AVB.

**Conclusions:**

Screening for G613A in *LMNA*/*C* in patients with lone AVB and their relatives might prevent sudden death in families affected by AVB but without familiarity for DCM. Lone AVB is an age-related disease caused by mutations in *LMN*A/C gene rather than a complication of DCM.

**Electronic supplementary material:**

The online version of this article (doi:10.1186/s12979-014-0019-3) contains supplementary material, which is available to authorized users.

## Background

Atrioventricular block (AVB) is a partial or complete block in the transmission of the electrical impulses originating in the atrium or sinus node and directed toward the AV node and ventricles. Depending on the gravity of AVB, cases are grouped into one of three different categories: type I AVB is characterized by prolongation of AV conduction time; type II AVB is characterized by failed transmission of some impulses, with consequent loss of some ventricular contractions; and type III AVB is characterized by complete AV dissociation and loss of many/all contractions or coordination with atrial contraction [1].

AVB can be associated with several cardiac disorders, such as dilated cardiomyopathy (DCM), ischemia, infarction, and fibrosis, and may present as an aggravation of existing cardiac suffering. AVB can also be caused by a highly heritable, primitive disease presenting itself at an early age [2]. Little is known about the origin of progressive cardiac conduction defects, such as congenital AVB, although linkage analyses of affected families of different nationalities has led to the identification of several causal mutations in sodium-channel, melastatin-4, and NK2 homeobox 5 genes, [3-5] and to the identification of a disease-related locus at chr1q32.2-q32.3 [6].

One of the most common pathologies associated with AVB is DCM, which is characterized by cardiac dilatation and reduced systolic function. In this case, onset has been correlated with several mutations in genes essential for the proper maturation of cardiomyocytes. For example, autosomal dominant DCM and conduction system diseases are linked to a disease locus at chr1p1-q21, where the gene for lamin A/C (*LMNA/C*) is encoded [7]. The coding region of *LMNA/C* spans approximately 24 kb, harbors 12 exons, and produces the alternative splice products LMNA (74 kDa) and LMNC (65 kDa) [8]. Lamins have an essential role in maintaining nuclear shape and structure, transcriptional regulation, nuclear pore function, and heterochromatin organization [9]. In patients with DCM associated with AVB and carriers of *LMNA*/*C* mutations, reduced LMNA/C expression caused damage to cardiomyocyte nuclei and nuclear membranes, such as focal disruptions, bleb formations, and nuclear pore clustering [10].

Apart from dilated cardiomyopathy 1A, *LMNA/C* mutations have also been linked to the premature aging syndrome Hutchinson’s progeria, skeletal myopathies (such as the autosomal dominant variant of Emery-Dreifuss muscular dystrophy and limb-girdle muscular dystrophy), Charcot-Marie-Tooth disorder type 2B1, mandibuloacral dysplasia, autosomal dominant partial lipodystrophy, and axonal neuropathy [9,11]. However, no clear genotype–phenotype relationship has been reported to date between *LMNA/C* mutations and cardiomyopathies [12]. Therefore, we decided to identify causative mutation(s) through exome sequencing of a family with an autosomal dominant form of AVB.

## Results and discussion

**Figure 1:**
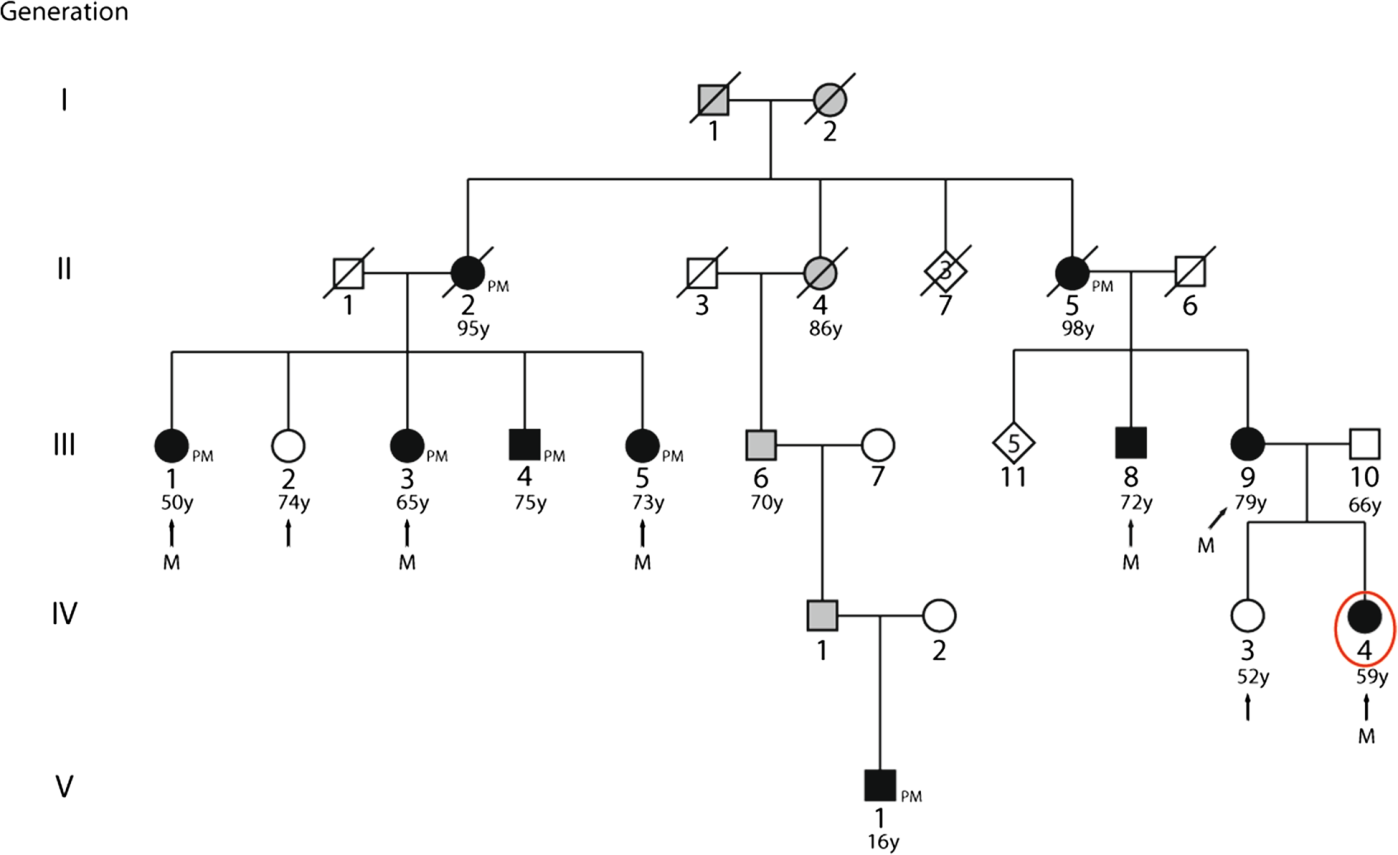
**Pedigree of the studied atrioventricular block family.** Generation is indicated with roman numerals, and individual ID is indicated with Arabic numerals. Solid squares (males) and circles (females) indicate affected subjects, open symbols indicate unaffected subjects, and gray symbols indicate unknown disease status. Diamonds indicate not-relevant subjects, and the numbers within them are the number of subjects. Diagonal lines indicate dead subjects. Arrows identify the analyzed subjects. M indicates the subjects carrying rs56816490. PM indicates pacemaker implantation. The proband is indicated with a red oval. Ages of subject are given beneath the individual’s ID number.

A large family from central Italy was recruited for the genetic study of a familial autosomal dominant form of lone, type II AVB. The pedigree was composed of 31 subjects belonging to five different generations, 10 of which presenting with the pathology. Six affected and five apparently unaffected members participated in the study (Figure 1). The criterion for AVB diagnosis was the presence of AV node block at either standard ECHO or 24-hour Holter recording, or the presence of an implanted pacemaker.

Electrophysiological study of the proband revealed a long AV-node refractory period of 420 msec at a cycle length of 600 msec, and 2:1 AV block at a cycle length of 450 msec. ECHO did not indicate the presence of DCM in the proband (see online Additional file 1), and there were no indications of DCM in the medical histories of any of the other affected family members, some of which had received a pacemaker (a summary of the subjects with known cardiovascular dysfunction is provided in Table 1). There was no significant information on predisposing life styles and risk factors for AVB in these subjects.

**Table 1:** **Known medical histories of subjects in the pedigree**

**Generation**	**Subject ID**	**Age**	**Gender**	**Medical history**
II	2	95	Female	Two pacemakers
II	5	98	Female	Pacemaker
II	7a	72	Male	Sudden death
II	7b	70	Male	Asthma
III	1	50	Female	Pacemaker
III	3	65	Female	Pacemaker
III	4	75	Male	Pacemaker
III	5	73	Female	Pacemaker
III	8	72	Male	Pacemaker
III	9	79	Female	Pacemaker
III	11a	66	Male	Sudden death
III	11b	75	Male	Sudden death
III	11c	71	Female	Small cardiac suffering
IV	4	59	Female	Pacemaker, atrial fibrillation
V	1	16	Male	Pacemaker

The exceptional longevity of some ancestors (95 years for II:1 and 98 years for II:5) and the reported [13] delay in the onset of age-associated diseases, including AVB, in long-living offspring induced us to run a study on only affected members, so as to avoid possible misclassification of affected status of the analyzed subjects [13].

**Figure 2:**
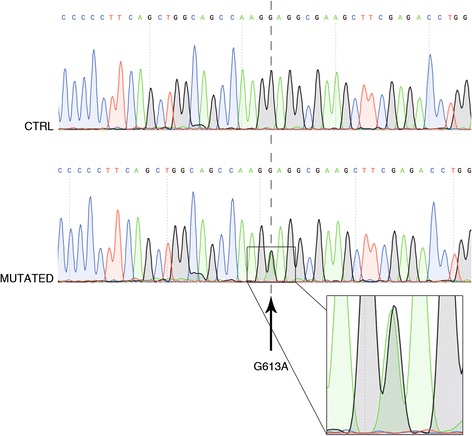
**Electropherogram of the lamin A/C gene.** Electropherograms of control (upper) and atrioventricular block-affected (lower) subjects showing heterozygosity for the *LMNA/C* mutation G613A in the latter. Heterozygosity is indicated by the presence of two peaks corresponding to G and A (arrow and magnification).

**Figure 3:**
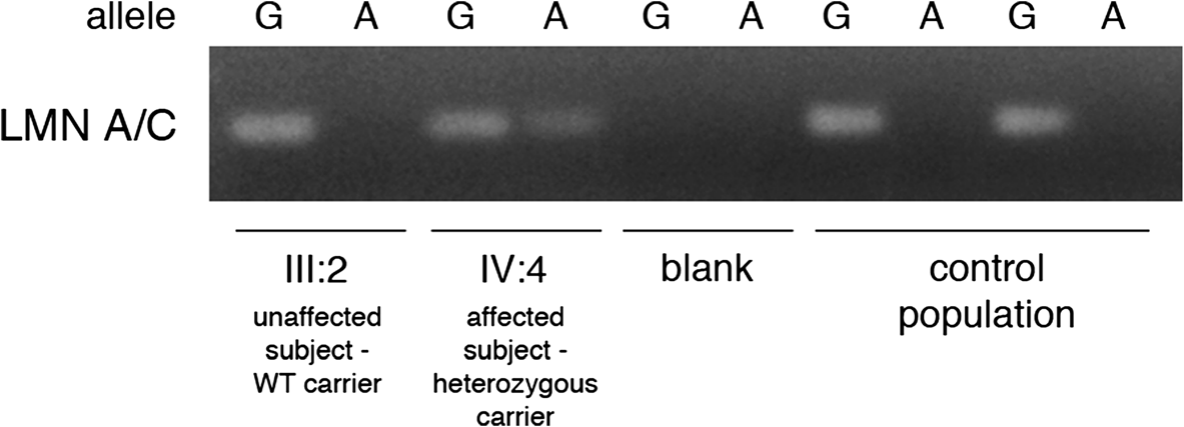
**Frequency analysis of controls with PCR for rs56816490.** Image of electrophoretic analysis of PCR validation amplifications of genomic DNA from pedigree subjects (III:2 and IV:4) and independent controls. Amplification of the mutated allele of *LMNA/C* (G613A) is present only in the affected subject (IV:4, the proband).

To identify the causative gene mutation(s), we sequenced the whole exome of three affected members of the family: one fourth-generation member (IV:4, the proband) and two third-generation subjects (III:5 and III:8). Filtered results indicated that 10 nonsynonymous and damaging mutations were common to the three subjects (Table 2). We then checked the presence of these mutations in three other affected family members (III:1, III:3, and III:9): out of the 10 mutations assessed, G613A in *LMN A/C* (rs56816490) was carried in a heterozygous fashion by all affected individuals (Figure 2). Analysis for the presence of rs56816490 in non-affected family members and in 100 control subjects, in agreement with the 1000 Genomes Project, [14] was negative (Figure 3).

**Table 2:** **Stop gain/nonsynonymous SNPs identified by exome sequencing**

**Variant coordinates**	**SNP ID**	**Gene**	**Function**	**Nucleotide changes**	MAF* (%)
chr1:111784045	-	*CHI3L2*	Nonsynonymous	G-- > A	
chr1:113162494	rs41283062	*CAPZA1*	Nonsynonymous	G-- > A	1.380
chr1:114367793	rs140698521	*PTPN22*	Nonsynonymous	C-- > T	0.064
chr1:156105704	rs56816490	*LMNA*	Nonsynonymous	G-- > A	
chr4:2744193	-	*TNIP2*	Nonsynonymous	A-- > G	
chr5:63802483	-	*RGS7BP*	Nonsynonymous	G-- > C	
chr10:11374607	-	*CELF2*	-	T-- > A	
chr11:104768141	rs138698464	*CASP12*	Nonsynonymous	G-- > A	1.335
chr21:34924550	rs142482063	*SON*	Nonsynonymous	G-- > T	0.044
chr22:19511925	rs885985	*CLDN5*	Stop gain	G-- > A	44.631

*,UCSC Genome Browser database. MAF = Minor allele frequency.

*LMNA*/C variations are responsible for many different diseases involving, above all, muscle tissue, including DCM with conduction defects [14]. However, to date there is no clear genotype–phenotype correlation between *LMNA/C* mutations and DCM associated with conduction disturbances. In the present study, we correlate the missense mutation G613A in *LMNA/C* (SNP ID rs56816490) with a familial, autosomal dominant form of AVB that we classify as “lone” based on the absence of DCM in the family’s medical history and on the absence of any abnormality in ventricular and atrial sizes in the proband. The SNP is located within the 6th exon of *LMNA*/*C*, which is located at chr1:156205704, and determines the amino acid change E317K in a segment of the LMNA/C coil 2 that does not interact with proteins or chromatin.

Arbustini et al. [15] have already described this mutation in a proband with AVB putatively associated with a late-onset DCM: the patient was suffering from AVB since age 44 years, had a family history of sudden death, and was implanted with pacemaker at age 69 years. The proband’s father, also a pacemaker carrier, received a post-mortem diagnosis of DCM, indicating that the son’s clinical status may worsen sometime in the future due to DCM, although this has not been documented to date [15]. Careful analysis of the mutations detected in DCM patients with conduction defects led Arbustini et al. [15] to conclude that clinical manifestations and disease evolution were more severe in patients with *LMNA*/*C* mutations that affected nuclear lamin interactions, whereas the E317K change, present in a non-interacting segment, was responsible for a mild phenotype.

In light of our data, we would re-consider the disease status of the LMNA/C E317K-carrying proband described by Arbustini et al. as affected with lone AVB. Of note, E317K is a rare change in LMNA/C, and both families were from Italy; thus, we cannot exclude a common origin of the mutation.

## Conclusions

Our results are important for genetic counselling, pointing to the need for screening G613A in *LMNA/C* in patients with lone AVB and their relatives. Indeed, in the family we investigated, three subjects (one in generation II, and two in generation III) had a medical history of sudden death, as did the E317K LMNA/C carriers reported by Arbustini et al. [15]. Furthermore, our data point to lone AVB as a disease caused by mutation of *LMNA/C* inducing accelerated aging, and thus makes appealing the hypothesis that AVB is an age-related disease on its own right, other than a complication of DCM.

## Methods

The study was conducted in accordance with the ethical principles that have their origins in the Declaration of Helsinki. Informed consent was obtained from all subjects in accordance with the Review Board rules of IRCCS Multimedica. Subjects were evaluated, when possible, by medical history, physical examination, and electrocardiography. Reports from echocardiography (ECHO), cardiac catheterization, electrophysiology studies, cardiac surgery, and/or autopsy were reviewed when available. Clinical assessment was performed without knowledge of genotype.

### DNA isolation and exome sequencing

Genomic DNA was isolated from whole blood, using the QIAamp DNA Blood Midikit (Qiagen), according to the manufacturer’s protocol. Exonic regions of genomic DNA of three affected subjects (IDs III:5, III:8, and IV:4) were enriched using either the TruSeq™ Exome Enrichment Kit (Illumina) or the Agilent Haloplex Exome kit based on DNA digestion and capture. Exomes were barcoded and sequenced at multiple sites on the Illumina HiSeq1000 platform, and either 2 × 76-bp (TruSeq) or 2 × 100-bp (Haloplex) PE libraries, using TruSeq SBS Kit v3–HS (Illumina) reagents and a TruSeq PE Cluster kit v.3-cbot-HS flow cell. Average coverage for all the experiments was 70x and at least 20x for 89% of the target. Paired sequencing reads were aligned to the reference genome (UCSC, hg19 build) using BWA [16] and sorted with SAMtools [17] and Picard (http://broadinstitute.github.io/picard/). Post-alignment processing (local realignment around insertions-deletions and base recalibration), SNV, and small insertions-deletions (ins-del) calling were performed with Genome Analysis Toolkit (GATK) [18] with parameters adapted to the haloplex-generated sequences. The called SNV and ins-del variants produced with both platforms were annotated using ANNOVAR [19].

### Data filtering

The results were first filtered to eliminate common variants (MAF > 1%), variants with low quality score, and variants not shared by all analyzed affected subjects, when covered. Additional frequency filters were used by comparing internal databases of whole exome sequencing data (n = 300). Prioritization was also made based on MAF frequency (complete data sets are available in online Additional file 2).

### Validation analysis

Genomic DNA of the remaining affected subjects was amplified by standard polymerase chain reaction (PCR) methods for selected single-nucleotide polymorphisms (SNPs). The amplified fragments were purified with the Wizard SV Gel and PCR Clean-Up System (Promega) and were sequenced in order to identify the mutation associated with AVB. Primer sequences and PCR conditions are given in Table 3.

**Table 3:** **Primer sequences used in the validation analysis**

**Gene**	**Forward Primer (5’-3’)**	**Reverse Primer (5’-3’)**	**Tm (°C)**
*CHI3L2*	TAACCCATTACTGACCCTCTCGT	CTGCTGGATCCCATCCTAGA	61
*CAPZA1*	GTGATTCCATCACTCGGCTT	GTCAGCATGGACCAAGAAGC	64
*PTPN22*	GACCCTATGGAGGCTCTGTTTA	AAATCAAGTAGAGGTTTTGCACTG	61
*LMNA*	CGAGGAGCTGCAGCAGTC	CGCATCTCGGCCATCTC	65.5
*TNIP2*	GAACCTGAGACCTGGCTTTGTG	CTGAAGCACTGCAGGCAGTG	65
*RGS7BP*	CCAGGGCAACAACCGG	GCAGTTTTCACCCACCATCTT	61
*CELF2*	TACTGACCAAATACCTAGCACC	AGAGGCTAGTCCTTTCCATC	61
*CASP12*	GCATTGCTCACCACAAACT	CGGTGTTCTGGTCCACAT	58
*SON*	GACCCTACAGGATAGCACCCA	CGCTCGTAGGCTGACATCAT	61
*CLDN5*	CCATGGCTAGAGGCGAGAC	GCACGGATTGGCTGCTT	61

### Mutation detection by PCR in controls

Two forward PCR primers were designed for the amplification of the rs56816490 locus in *LMNA*/C: a primer targeting wild-type *LMNA/C* (5′-CAGCTGGCAGCCAAGg-3′) and another primer targeting a mutated variant (5′-CAGCTGGCAGCCAAGa-3′). Direct amplification of genomic DNA of 100 controls was performed using independently both the forward primer and the reverse primer (5′-CGCATCTCGGCCATCTC-3′) followed by visualization on an agarose gel with ethidium bromide staining.

## Additional files

Additional file 1:
**Additional figure: cardiac assessment of the Proband. Recent echocardiography and electrocardiography of the proband.**
Additional file 2:
**Complete data sets: the file lists the results of exome sequencing analysis.**

## Abbreviations

AVB: Atrioventricular block
DCM: Dilated cardiomyopathy
ECHO: Echocardiogram
LMNA/C: Lamin A/C
PCR: Polymerase chain reaction
SNP: Single-nucleotide polymorphism
