# Supplementary material for: A G613A missense in the Hutchinson’s progeria lamin A/C gene causes a lone, autosomal dominant atrioventricular block
Source: Immun Ageing. 2014 Nov 26;11:19. doi: 10.1186/s12979-014-0019-3 (PMC4251685; doi:10.1186/s12979-014-0019-3)
Supplement: Additional file 1: — Additional figure: cardiac assessment of the Proband. Recent echocardiography and electrocardiography of the proband. [file 12979_2014_19_MOESM1_ESM.pdf]

ADDITIONAL FIGURE

A

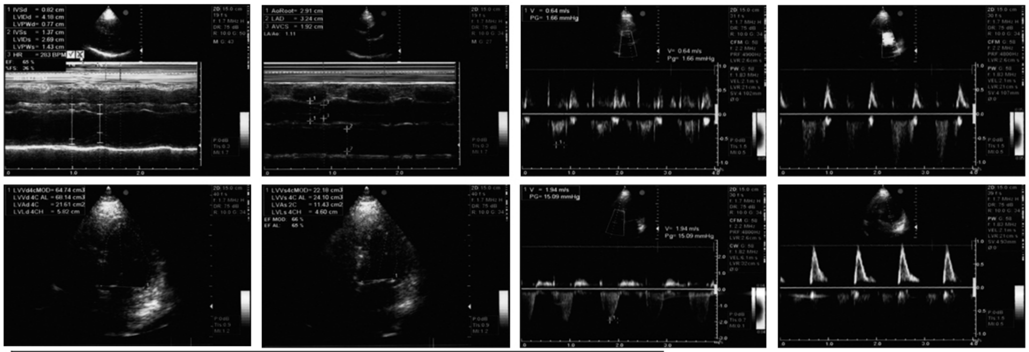

exam		value
Interventricular septal end diastolic dimension	IVSd	0,99 cm
Left Ventricle Internal diastolic Dimension	LVIDd	4,51 cm
Left Ventricle Posterior Wall diastolic Dimension	LVPWd	1,04 cm
Interventricular septal end systolic dimension	IVSs	1,43 cm
Left Ventricle Internal systolic Dimension	LVIDs	2,86 cm
Left Ventricle Posterior Wall systolic Dimension	LVPWs	1,59 cm
Heart rate	HR	51 bpm
Eject fraction	EV	67%
Fractional shortening	FS	37%
Aortic root diameter	AoRoot	2,91 cm
Left atrial dimension	LAD	3,40 cm
Left atrial/aortic root dimension ratio	LA/Ao	1,11
Left Ventricular Volume in Diastole MOD	LVVd MOD	64,73 cm^3
Left Ventricular Volume in Diastole AL	LVVd AL	68,14 cm^3
Left Ventricular Area in Diastole	LVAd	21,61 cm^2

B

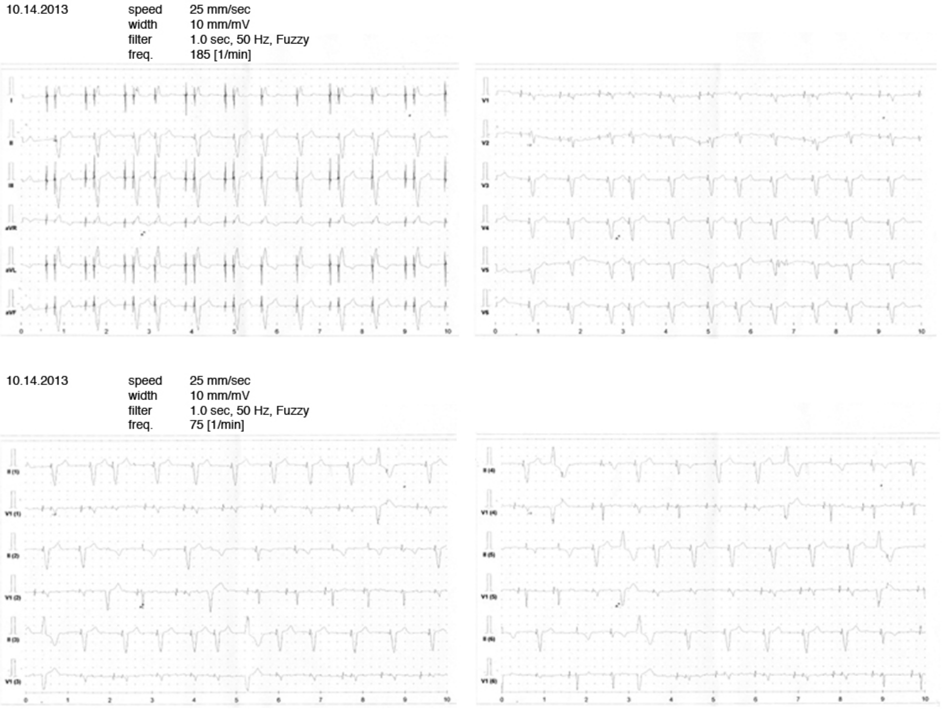

Additional Figure. Cardiac assessment of the Proband. Recent echocardiography (A) and electrocardiography (B) of the proband, demonstrating the absence of dilated cardiomyopathy in the subject.
